# Syncing the brain’s networks: dynamic functional connectivity shifts from temporal interference

**DOI:** 10.3389/fnhum.2024.1453638

**Published:** 2024-10-29

**Authors:** Zhiqiang Zhu, Dongsheng Tang, Lang Qin, Zhenyu Qian, Jie Zhuang, Yu Liu

**Affiliations:** ^1^School of Kinesiology, Shenzhen University, Shenzhen, China; ^2^School of Kinesiology, Shanghai University of Sport, Shanghai, China

**Keywords:** temporal interference, non-invasive brain stimulation, dynamic functional connectivity, resting-state fMRI, primary motor cortex

## Abstract

**Background:**

Temporal interference (TI) stimulation, an innovative non-invasive brain stimulation approach, has the potential to activate neurons in deep brain regions. However, the dynamic mechanisms underlying its neuromodulatory effects are not fully understood. This study aims to investigate the effects of TI stimulation on dynamic functional connectivity (dFC) in the motor cortex.

**Methods:**

40 healthy adults underwent both TI and tDCS in a double-blind, randomized crossover design, with sessions separated by at least 48 h. The total stimulation intensity of TI is 4 mA, with each channel’s intensity set at 2 mA and a 20 Hz frequency difference (2 kHz and 2.02 kHz). The tDCS stimulation intensity is 2 mA. Resting-state functional magnetic resonance imaging (rs-fMRI) data were collected before, during, and after stimulation. dFC was calculated using the left primary motor cortex (M1) as the region of interest (ROI) and analyzed using a sliding time-window method. A two-way repeated measures ANOVA (group × time) was conducted to evaluate the effects of TI and tDCS on changes in dFC.

**Results:**

For CV of dFC, significant main effects of stimulation type (*P* = 0.004) and time (*P* < 0.001) were observed. TI showed lower CV of dFC than tDCS in the left postcentral gyrus (*P* < 0.001). TI-T2 displayed lower CV of dFC than TI-T1 in the left precentral gyrus (*P* < 0.001). For mean dFC, a significant main effect of time was found (*P* < 0.001). TI–T2 showed higher mean dFC than tDCS-T2 in the left postcentral gyrus (*P* = 0.018). Within-group comparisons revealed significant differences between time points in both TI and tDCS groups, primarily in the left precentral and postcentral gyri (all *P* < 0.001). Results were consistent across different window sizes.

**Conclusion:**

20 Hz TI stimulation altered dFC in the primary motor cortex, leading to a significant decreasing variability and increasing mean connectivity strength in dFC. This outcome indicates that the 20 Hz TI frequency interacted with the motor cortex’s natural resonance.

## 1 Introduction

Noninvasive brain stimulation (NIBS), such as transcranial electrical stimulation (tES), has emerged as a powerful tool in neurorehabilitation (Hummel and Cohen, 2006; Kang et al., 2016; Weightman et al., 2020; Cinosi et al., 2021; Zaehle, 2021). This method aims to modulate brain function non-invasively, targeting specific neural circuits to improve outcomes. By enhancing brain function and refining motor control, NIBS holds promise for treating a variety of neurological and functional disorders (Nitsche and Paulus, 2000). For individuals suffering from motor impairments, by stimulating neural circuits involved in movement, these techniques facilitate motor recovery and augment motor learning processes. This approach offers promising therapeutic benefits for conditions that impair motor function (Reis and Fritsch, 2011). However, the complexity of the human brain brings significant challenges for the application of NIBS, particularly in terms of its spatial resolution limitations and the less precision in targeting specific brain regions (Vöröslakos et al., 2018; Toth et al., 2024). Therefore, enhancing tES’s penetration and focality are key to improving its effectiveness.

Grossman et al. developed a novel noninvasive brain stimulation called “Temporal Interference” (TI) (Grossman et al., 2017). This approach forms a low-frequency envelope wave by applying two high-frequency alternating currents with a slight frequency difference. High-frequency electric fields correspond to shorter wavelengths. According to the propagation characteristics of electromagnetic waves, the shorter the wavelength, the stronger the penetration capability of the electric field (Huang and Wang, 2015). Moreover, this envelope enables brain tissue to respond specifically to the low frequency while remaining unresponsive to the high-frequency components. As a result, the low-frequency envelope can penetrate deeper into the brain, allowing for precise targeting of specific neural populations without impacting the surrounding tissue (Grossman et al., 2017). The quantify data showed that TI can modulate neural activity at depths exceeding 50mm beneath the cortical surface (Violante et al., 2023). In contrast, tDCS primarily affects superficial cortical areas within 10–20 mm due to rapid attenuation in deeper tissues (Bikson et al., 2010). Moreover, at different target depths, Wang M. et al. (2023) revealed that TI enhances focality by reducing the activated volume outside the target by 60%. The existing body of evidence indicates that TI may yield effects that surpass those of tDCS. However, factors such as individual variations in cortical excitability (Wiethoff et al., 2014), skull thickness (Opitz et al., 2015), precise electrode placement (Bikson et al., 2010), and the cognitive state of participants during stimulation sessions (Li et al., 2015) may significantly influence the observed outcomes.

Currently, much research on TI stimulation has demonstrated the effectiveness of TI (Rampersad et al., 2019; Mirzakhalili et al., 2020; Esmaeilpour et al., 2021). Animal experiments in rodents and non-human primates have validated the spatial precision and neuromodulatory effects of TI (Acerbo et al., 2022; Carmona-Barrón et al., 2023; Kwak et al., 2023). Cadaver studies have confirmed the ability of TI stimulation to penetrate deep into the brain tissue without the need for invasive procedures (Acerbo et al., 2022; Violante et al., 2023). Preliminary human trials have also provided promising results, suggesting the potential of TI stimulation for therapeutic application (Ma et al., 2021; Zhang et al., 2022; Wessel et al., 2023). At the same time, our study found that TI effectively enhanced the functional connectivity strength between the primary and secondary motor cortex, comparable to the effects of tDCS (Zhu et al., 2022). To some extent, it suggested that TI may have better neuromodulatory effects to tDCS in enhancing cortical excitability and motor function. However, the mechanisms through which TI affects dynamic functional connectivity remain unclear.

Dynamic variation in functional connectivity refers to the temporal fluctuations observed in functional connectivity, quantifiable through specific metrics such as variance, entropy, and state transitions. Variance serves to evaluate the extent of fluctuation within the functional connectivity matrix (Allen et al., 2014), while entropy quantifies the complexity and uncertainty inherent in functional connectivity states (Blair et al., 2022). State transition analysis examines the frequency of transitions between these states, providing insights into the evolving patterns of connectivity over time (Jiang et al., 2022). By employing these metrics, researchers can attain a more nuanced understanding of the stimulation effects on dFC. Unlike static functional connectivity (sFC), which examines the overall correlation of brain regions over an entire experimental session. dFC investigates the dynamic changes of functional connectivity between brain regions on short time scales, revealing the constant changes of brain functional states over time (Polanía et al., 2011; Shine et al., 2016; Allen et al., 2018). Moreover, it is also closely related to various biophysical mechanisms, such as synaptic plasticity or neural oscillation. Alterations in synaptic plasticity led to adjustments in neuronal communication modes and the efficiency of information transmission. These micro-level changes manifest at the macro scale as dynamic alterations in the brain’s functional connectivity patterns—that is, changes in dFC (Deco et al., 2011). Additionally, dFC is also related to brain oscillations because oscillatory activity modulates neuronal synchrony, influencing the dynamic changes in functional connectivity between brain regions (Buzsáki and Draguhn, 2004; Fries, 2005). Oscillations at different frequencies coordinate neural network activities at various scales, allowing for flexible information transmission and integration within the brain (Siegel et al., 2012). Variations in oscillation amplitude and phase can lead to the strengthening or weakening of functional connections, reflected in the dynamic patterns of dFC (Hutchison et al., 2013). This dynamic perspective is particularly important, as the brain is a complex and highly dynamic system, constantly reorganizing its functional connections in response to internal and external stimulation. By capturing these temporal changes in functional connectivity, researchers can gain a more comprehensive understanding of the brain’s functional organization and information processing mechanisms (Damaraju et al., 2014; Rashid et al., 2014; Sourty et al., 2016). In analyzing dFC, selecting the appropriate window size is crucial (Zhang et al., 2018). Although no studies directly compare specific time windows with neural dynamics, previous studies had confirmed that the window length of 30 TR can better reflect the dynamic characteristics of the brain (Wang Y. et al., 2023), particularly the window length under 40 seconds, significantly enhance test-retest reliability (Zhang et al., 2018).

Therefore, the aim of this study is to investigate the effects of TI by comparing it with tDCS on the motor cortex in healthy adults, exploring the dynamic mechanisms through which TI stimulation influences FC. The hypothesis is that TI will decrease the dynamic variation of functional connectivity, enhance dynamic stability in the motor cortex, and achieve greater efficiency in these effects compared to tDCS.

## 2 Materials and methods

### 2.1 Participants

40 healthy adults participated in the study, comprising 31 males with a mean age of 25.97 ± 3.53 years and 9 females with a mean age of 24.11 ± 0.93 years. All participants were right-handed, verified by the Edinburgh Handedness Inventory (Oldfield, 1971). Inclusion criteria for the study included: (1) being aged between 18 and 35 years; (2) having no history of neurological disorders, medication use, or metal implants; and (3) experiencing no adverse reactions to non-invasive brain stimulation.

Before commencing the experiment, participants underwent a familiarization session to grow accustomed to the stimulation and protocol. The study was designed to follow ethical conduct, with informed consent obtained from all participants, in conformity with Helsinki Declaration. Following exclusions due to excessive motion artifacts, 32 participants remained in the final analysis. The study protocol received approval from the Shanghai University of Sport’s Institutional Review Board (102772020RT116) and was registered with the Chinese Clinical Trial Registry (ChiCTR2100052866).

### 2.2 Experimental design

This study utilized a randomized, double-blind, crossover design, with participants randomly assigned to either the TI or tDCS stimulation through computer-generated random numbers. Furthermore, stratified randomization was implemented based on gender to ensure balanced representation across both stimulation conditions. All participants underwent testing within the same time frame to control for baseline connectivity, and they were instructed to refrain from vigorous activities and stimulants, including alcohol and coffee, for 8 h prior to the experiment. Importantly, the personnel responsible for managing the randomization process did not engage in data processing or testing, thereby further minimizing potential bias. The two sessions were separated by a minimum interval of 48 h to ensure adequate washout of any stimulation effects.

**FIGURE 1:**
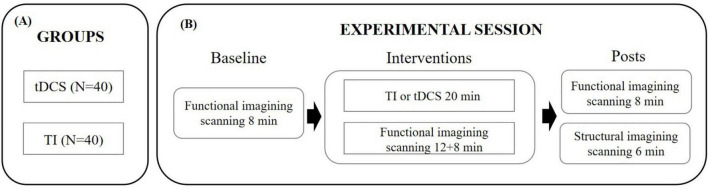
Experimental protocol. **(A)** Assignment of participants to one of the experimental groups. **(B)** Timeline of the procedures accomplished before, during and after the tES-MRI protocol. TI, temporal interference stimulation; tDCS, transcranial direct current stimulation.

Each study visit consisted of two imaging sessions: a functional scan and a structural scan. The functional magnetic resonance imaging (fMRI) scan monitored brain activity at rest for 8 min, followed by 20 min of brain stimulation, and concluded with an additional 8 min of rest. This was followed by a 6-min structural imaging scan to acquire anatomical data. The experimental design is illustrated in Figure 1.

### 2.3 Brain stimulation parameters and session procedures

#### 2.3.1 TI

**FIGURE 2:**
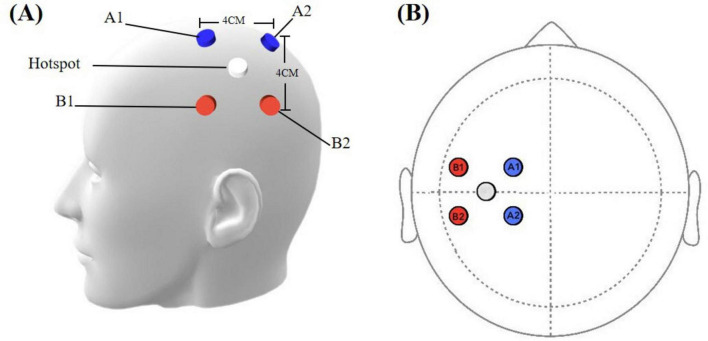
**(A)** Simulation head model with electrode placements. **(B)** Spatial configuration of stimulation electrodes. Blue electrodes: cathode, 2000 Hz channel; red electrodes: anode, 2020 Hz channel. White circle indicates the hot spot.

The subcortical area beneath “hotspot” of the left first dorsal interosseous (FDI) muscle is served as the target of TI stimulation (Soterix Medical, New Jersey, USA). It was defined as the scalp location where transcranial magnetic stimulation (TMS) pulses consistently elicited motor evoked potentials (MEPs) with the highest peak-to-peak amplitude in the contralateral FDI muscle, as measured by surface electromyography (EMG). The hotspot was identified by applying single-pulse TMS, utilizing a figure-of-eight coil oriented at a 45°angle to the mid-sagittal line. Initial stimulator output was set to 70% of maximum. Starting from C3, the coil was moved in 0.5 cm steps anteroposteriorly and mediolaterally, while gradually decreasing intensity by 5% steps, to find positions eliciting MEPs in the FDI muscle. When points were found where MEPs above 100 μV could not be elicited, the intensity was further decreased in 1–2% steps. The search process was repeated iteratively until MEPs were observed in 3 out of 3 trials at a given position, while stimulation of adjacent positions did not evoke reliable MEPs in 3 trials. If no MEPs were evoked at any position at a given intensity, while at an intensity 1% higher, 3 MEPs were still observed out of 3 trials in more than one point, the ‘hot spot’ was defined as the position in which the largest mean MEP amplitude was detected (Conforto et al., 2004). The “O” point depicted in Figure 2 is defined as the “hotspot” for the first dorsal interosseous muscle (FDI). The subcortical area beneath the “O” point serves as the target for TI stimulation. Based on point “O”, four electrodes were positioned parallel to the line connecting the eyebrow center and occipital tuberosity. Four electrodes were placed at positions A1, A2, B1, and B2, with a 4 cm distance between each pair (A1-A2, A1-B1, B1-B2, A2-B2) (Figure 2). The A1-A2 channel was operated at 2000 Hz, while the B1-B2 channel was at 2020 Hz, resulting in a 20 Hz frequency difference. The current intensity was 2 mA per channel, for a total of 4 mA (peak-peak). The stimulation duration is 20 min, with two short periods of 30s ramp-up and ramp-down stimulation.

#### 2.3.2 tDCS

**FIGURE 3:**
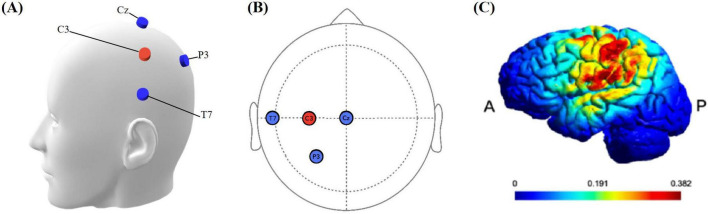
**(A)** Simulation head model with electrodes. The blue electrode is the cathode, and the red electrode is the anode. **(B)** Location of stimulation electrodes. Based on the international 10–20 system, anodal (C3), cathodal (T7, P3, Cz). **(C)** Simulated electrical field. P: Posterior, A: anterior. The color bar is the intensity of stimulation.

The tDCS protocol employed the MR-compatible DC-STIMULATOR PLUS device (NeuroConn, Ilmenau, Germany), with specific settings as described in Esmaeilpour et al. (2020). Four MRI-compatible rubber electrodes (1.5 cm × 2 cm) were used to deliver continuous direct current. Each electrode had a resistance of less than 30KΩ. The STIMWEAR software allowed for online target editing for neuroelectric stimulation, enabling us to define the target area as the left FDI-M1. Stimulation parameters were set to a maximal current intensity of 2 mA, distributed strategically across the electrodes based on the 10–20 EEG system: C3 received 2000 μA (the anodes of the three electrode pairs were inserted into the C3 silicone electrode), while P3, T7, and Cz were set at −774 μA, −684 μA, and −542 μA, as depicted in Figure 3. The protocol comprised a continuous 20-min stimulation period at the defined intensity, flanked by 30-second ramp-up and ramp-down.

### 2.4 MRI data acquisition

Neuroimaging data were acquired using a 3.0 Tesla Siemens MAGNETOM Prisma whole-body MRI system (Siemens Healthcare, Erlangen, Germany) equipped with a 64-channel phased-array head coil for radio frequency (RF) reception and transmission. All subjects received earplugs to protect them from the noise of the head coil. They were instructed to remain awake, still, and focused on a fixation cross with open eyes, avoiding directed thoughts. Eye states were continuously monitored externally. Blood oxygen level-dependent (BOLD) signals were obtained using gradient-echo echo-planar imaging (EPI) sequences with the following parameters: repetition time (TR) = 1000 ms, echo time (TE) = 30 ms, flip angle = 100°, field of view (FOV) = 240 × 240 mm^2^, voxel size = 3 × 3 × 3 mm^3^, 48 contiguous oblique axial slices parallel to the AC-PC line, and simultaneous multislice acquisition. Three functional runs were performed. The first and third runs consisted of 488 brain volumes each, lasting 8 min and 8 s. The second run acquired 1268 brain volumes over 21 min and 8 s. High-resolution structural images were collected using a 3D MP2RAGE (magnetization-prepared 2 rapid acquisition gradient echoes) sequence, with the following parameters: TR = 3130 ms, TE = 2.98 ms, flip angle = 12°, FOV = 256 × 256 mm^2^, voxel size = 1 × 1 × 1 mm^3^, 176 sagittal slices covering the whole brain.

### 2.5 Data processing

#### 2.5.1 MRI data pre-processing

Brain imaging data were preprocessed with the Data Processing Assistant for Resting-State fMRI [DPABI V5.4 (Yan and Zang, 2010)],^1^ SPM12,^2^ which are based on MATLAB). The imaging data was initially converted from DICOM to NIFTI format. To maintain consistency across all time points (T1, T2, and T3), we employed a standardized duration of 488 seconds for each analysis, analyzing the entire duration for T1 and T3, while for T2, we concentrated on the final 488 seconds of the 1208 seconds stimulation period. The initial 10 time points (corresponding to 10 seconds, with a TR of 1000 ms) of functional data were excluded to mitigate the effects of initial magnetic field instability and to facilitate participant acclimatization. The volumes underwent pre-processing, including slice timing correction, motion correction, and co-registration with high-resolution T1-weighted images. Participants with excessive head motion, defined as greater than 2.0 mm in translation or exceeding 2.0° in rotation (calculated on a frame-wise basis), were excluded from the analysis (Chen et al., 2018; Li et al., 2023). Of the initial 40 participants, 8 were excluded due to excessive head motion, yielding a final sample of 32 participants for subsequent analysis. Following motion correction, the structural image was co-registered with the mean functional image, and then partitioned into gray matter, white matter, and cerebrospinal fluid compartments. Finally, the segmented images were normalized to MNI space using the DARTEL algorithm, facilitating group-level analyses (Ashburner, 2007). Subsequently, the motion-corrected functional volumes were standardized to the MNI template space using the transformation matrices derived from their respective structural images, and resampled to a uniform voxel size of 3 mm × 3 mm × 3 mm. The voxel size of 3 mmł is fairly standard in neuroimaging research, but it may carry the risk of obscuring some fine-grained cortical activations. To minimize the impact of confounding variables, the method of Friston 24 was employed for head motion correction. These methods include 3 translational parameters (x, y, z) and 3 rotational parameters (roll, pitch, yaw), along with their temporal derivatives and squared terms (Friston et al., 1996). The signals from CSF and white matter (nuisance regressors) based on anatomical segmentations derived from structural MRI images (Zhang et al., 2001). Furthermore, linear detrending and band-pass filtering (0.01–0.08 Hz) were applied to mitigate the effects of low-frequency drift and high-frequency physiological noise. Notably, the use of global signal regression remains a topic of ongoing debate and was thus not employed in the present analysis (Murphy et al., 2009), Instead, we opted to preserve the global signal in our analysis. Finally, to mitigate the effects of imperfect normalization and enhance the signal-to-noise ratio, we applied a spatial smoothing procedure using a 6-mm full-width half maximum isotropic Gaussian kernel, thereby reducing the impact of noise and improving the overall data quality.

#### 2.5.2 Calculation of dFC

To analyze dynamic functional connectivity (dFC), the Toolkits for Temporal Dynamic Analysis (TDA) were utilized (Yan et al., 2017). The process commenced with defining the region of interest (ROI) for the FDI in primary motor cortex (FDI-M1), based on MNI coordinates (−36, −24, 54) reported by Yao et al. (2016). A spherical ROI with a 5mm radius was generated around these coordinates in the extraction interface for ROI. Subsequently, A Hamming window was applied in a sliding manner to the whole-brain BOLD signal time series. A dynamic analysis was performed using a sliding window approach with a window size of 30 TR and a step size of 1 TR, generating 449 windows from the 478-time interval dataset (Zalesky and Breakspear, 2015; Liu et al., 2017). To enhance the robustness of our findings, we conducted a repeated analysis using alternative window widths of 25 and 35 TR. This allowed us to meticulously record the functional connectivity strength and patterns across each scenario. The results revealed that the overall trends and main findings remained consistent throughout the analyses. In this process, the Pearson’s correlation coefficient (*r*-value) was calculated between this seed region’s mean time series and that of each voxel in the whole-brain gray matter mask, capturing dFC changes over time. The resulting *r*-values were then converted to z-scores using Fisher’s transformation. Moreover, two complementary metrics were calculated: the mean of dFC and the coefficient of variation (CV) of dFC. The mean of dFC was computed by averaging zFC values across all time windows for each voxel, thereby representing overall connectivity strength. The CV of dFC was calculated as the standard deviation of zFC divided by its mean across time windows, thus quantifying the relative temporal variability in connectivity.

### 2.6 Statistical analysis

Whole-brain voxel-wise analyses were performed using SPM12 (Wellcome Trust Centre for Neuroimaging, London, UK) implemented in MATLAB 2020a (MathWorks, Natick, MA, USA). To examine differences in dFC between TI and tDCS, we employed a two-way repeated measures ANOVA (2 × 3) with factors ‘group’ (TI, tDCS) and ‘time’ (baseline, online-stimulating, post-stimulating). Consistent with previous studies, an anatomical mask of the left precentral and postcentral regions, obtained from the Automated Anatomical Labeling (AAL) atlas, was used to extract the mean t-value for each cluster that met the minimum thresholds (5% alpha level; 2-voxel minimum) (Szaflarski et al., 2024). We investigated the interaction effect between group and time, as well as the main effects of group and time. Post hoc analyses were conducted for clusters showing significant main effects. A voxel-wise threshold of *p* < 0.005 was applied, followed by a false discovery rate (FDR) correction of *p* < 0.05 at the cluster level to correct for multiple comparisons. The corresponding cluster size threshold for analyses of the CV of dFC and the mean of dFC was 5 and 30 voxels, respectively. Peak and subpeak coordinates of significant clusters are reported in standard MNI space. Regional identification was performed using the AAL atlas and Brodmann templates, as implemented in MRIcron.^3^

## 3 Results

For CV of dFC, there was no significant time × stimulation-type interaction effect (P > 0.05, cluster-level FDR corrected). A significant main effect of the stimulation type for CV of dFC was found (*P* = 0.004, cluster-level FDR corrected). Additionally, a significant main effect of time for CV of dFC was found (*P* < 0.001, cluster-level FDR corrected).

**FIGURE 4:**
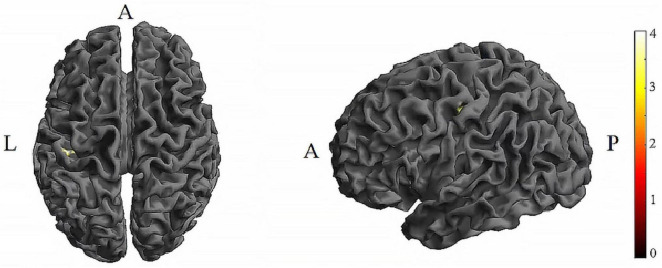
Significant differences in brain regions for the CV of dFC between tDCS-T2 and TI-T2. Compared with tDCS-T2, TI-T2 showed significantly increased CV of dFC (voxel *p* < 0.005, cluster *p* < 0 .05, cluster-level FDR corrected, cluster size ≥ 31 voxels). The color bar indicates the *t*-value. CV, Coefficient of Variation; T2, during stimulation phase; A, anterior; L, left; P, posterior.

**FIGURE 5:**
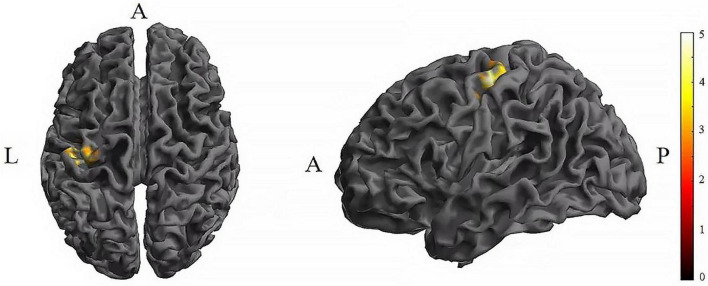
Significant differences in brain regions for the CV of dFC between TI-T1 and TI-T2. Compared with TI-T1 group, TI-T2 showed significantly increased CV of dFC (voxel p < 0.005, cluster *p* < 0 .05, cluster-level FDR corrected, cluster size ≥ 91 voxels). The color bar indicates the *t*-value. CV: Coefficient of Variation; T1, baseline phase; T2, during stimulation phase; A, anterior; L, left; P, posterior.

Further pairwise comparisons revealed that (1) in stimulation-type pairwise comparisons, the TI showed lower CV of dFC than the tDCS in the left postcentral (*P* < 0.001, cluster-level FDR corrected). The cluster size was 31 voxels. No significant differences were observed in other between-group pairwise comparisons (Table 1 and Figure 4). (2) In stimulation-phase pairwise comparisons, the TI-T2 displayed lower CV of dFC than the TI-T1 in the left precentral (*P* < 0.001, cluster-level FDR corrected). The cluster size was 91 voxels (Table 1 and Figure 5). No significant differences were found in other within-group pairwise comparisons. With respect to CV of dFC between TI and tDCS, the results obtained with a window size of 25 or 35 TR were similar to those achieved using a window size of 30 TR (Supplementary Appendix 1).

**TABLE 1:** Brain regions with Significant differences in CV of dFC using a 30 TR sliding window.

Comparisons	Brain regions/BA	Peak MNI coordinates			Cluster voxels	Peak *t*-values
		x	y	z		
Interaction effects	–	–	–	–	–	–
The main effect of group	Postcentral, L/3	−48	−24	51	13	3.19
The main effect of time	Precentral, L/6	−42	−15	54	47	4.07
tDCS−T2 vs TI−T2	Postcentral, L/4	−42	−24	57	30	3.57
TI−T1 vs TI−T2	Precentral, L/4	−42	−15	54	85	4.45

BA, Brodmann’s area, L, left; T1: baseline; T2: during stimulation; T3: after stimulation.

For mean of dFC, there was no significant time × stimulation-type interaction effect (P = 0.676, cluster-level FDR corrected). The analysis did not reveal a significant main effect of stimulation type on mean dFC (*P* = 0.053, cluster-level FDR corrected). Additionally, a significant main effect of time for mean of dFC was found (*P* < 0.001, cluster-level FDR corrected).

**FIGURE 6:**
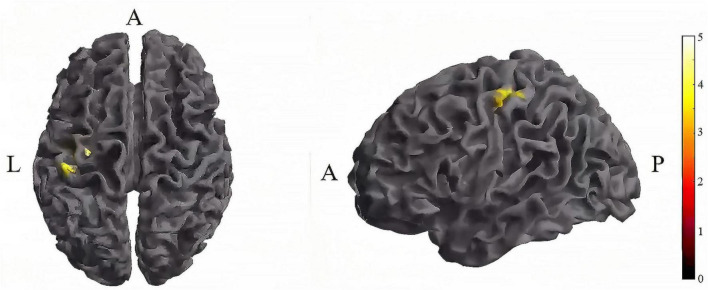
Significant differences in brain regions for mean of dFC between tDCS-T2 and TI-T2. Compared with tDCS-T2, TI-T2 showed significantly increased mean of dFC (cluster *p* < 0 .05, cluster-level FDR corrected, cluster size ≥ 84 voxels). The color bar indicates the t-value. T2, during stimulation phase; A, anterior; L, left; P, posterior.

**FIGURE 7:**
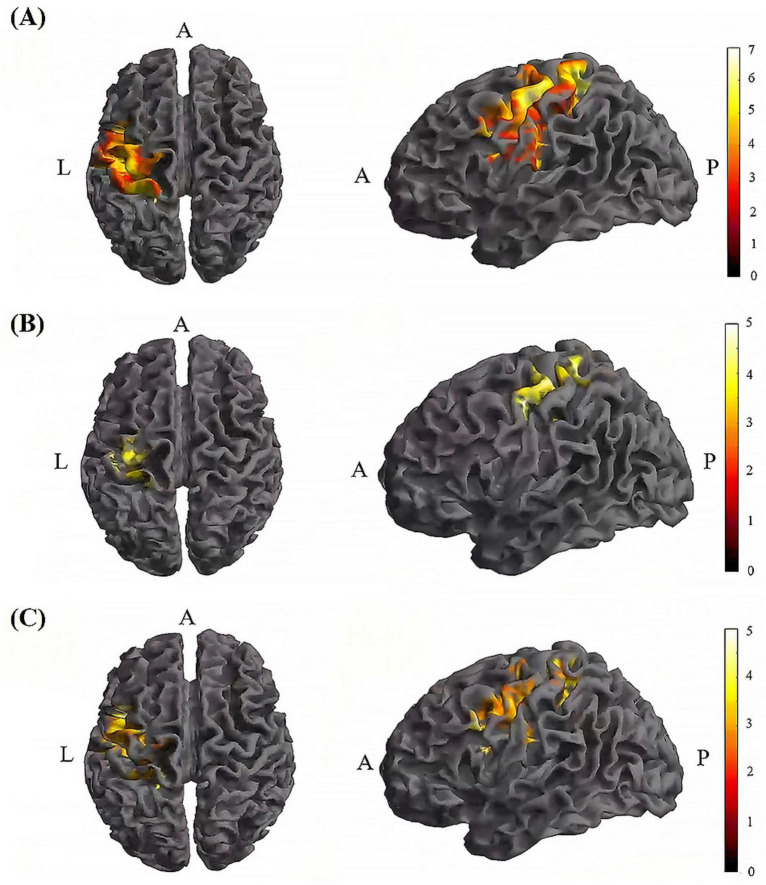
Significant differences in brain regions for mean of dFC within the TI group. **(A)** Brain regions with significant difference in mean of dFC between TI-T2 and TI-T1. Compared with TI-T1 group, TI-T2 group showed significantly increased mean of dFC. **(B)** Brain regions with significant difference in mean of dFC between TI-T3 and TI-T1. Compared with TI-T1, TI-T3 showed significantly increased mean of dFC. **(C)** Brain regions with significant difference in mean of dFC between TI-T2 and TI-T3. Compared with TI-T3, TI-T2 showed significantly increased mean of dFC. The color bar indicates the t-value. T1, baseline phase; T2, during stimulation phase; T3, post stimulation phase; A, anterior; L, left; P, posterior.

**FIGURE 8:**
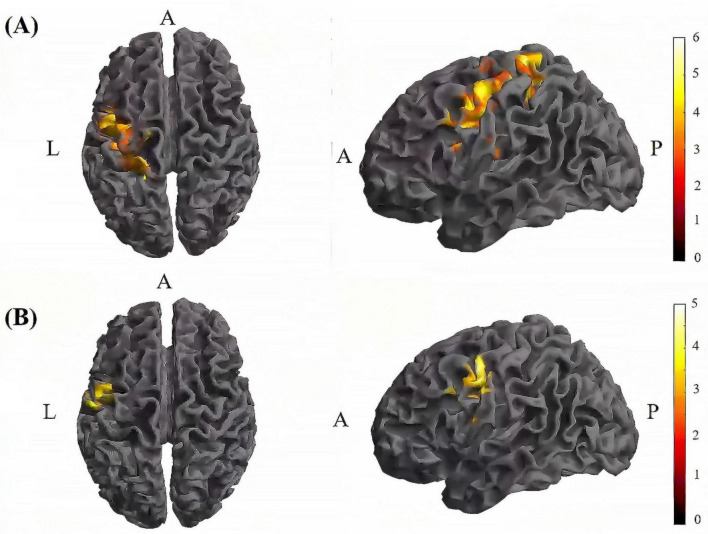
Significant differences in brain regions for mean of dFC within the tDCS group. **(A)** Brain regions with significant difference in mean of dFC between tDCS-T2 and tDCS-T1. Compared with tDCS-T1, tDCS-T1 showed significantly increased mean of dFC. **(B)** Brain regions with significant difference in mean of dFC between tDCS-T2 and tDCS-T3. Compared with tDCS-T3, tDCS-T2 showed significantly increased mean of dFC. The color bar indicates the *t*-value. T1, the baseline phase; T2, during stimulation phase; T3, post stimulation phase; A, anterior; L, left; P, posterior.

Further pairwise comparisons revealed that (1) in stimulation-type pairwise comparisons, the TI-T2 showed higher mean of dFC than the tDCS-T2 in the left postcentral (*P* = 0.018, cluster-level FDR corrected). The cluster size was 84 voxels. No significant differences were observed in other between-group pairwise comparisons (Table 2 and Figure 6). (2) In stimulation-phase pairwise comparisons, in the TI group, the TI-T2 displayed higher mean of dFC than the TI-T1 in the left precentral (*P* < 0.001, cluster-level FDR corrected). The cluster size was 1471 voxels. The TI-T3 displayed higher mean of dFC than the TI-T1 in the left precentral (*P* < 0.001, cluster-level FDR corrected). The cluster size was 224 voxels. The TI-T2 displayed higher mean of dFC than the TI-T3 in the left precentral (*P* < 0.001, cluster-level FDR corrected). The cluster size was 630 voxels (Table 2 and Figure 7). In the tDCS group, the tDCS-T2 displayed higher mean of dFC than the tDCS-T1 in the left postcentral (*P* < 0.001, cluster-level FDR corrected). The cluster size was 838 voxels. The tDCS-T2 displayed higher mean of dFC than the tDCS-T1 in the left precentral (*P* < 0.001, cluster-level FDR corrected). The cluster size was 264 voxels. No significant differences were found in other within-group pairwise comparisons (Table 2 and Figure 8).

**TABLE 2:** Brain regions with significant differences in mean of dFC using a 30 TR sliding window.

Comparisons	Brain regions/BA	Peak MNI coordinates			Cluster voxels	Peak *t*-values
		x	y	z		
Interaction effects	–	–	–	–	–	–
The main effect of group	–	–	–	–	–	–
The main effect of time	Postcentral, L/4	−21	−30	69	1433	6.61
TI–T2 vs tDCS–T2	Postcentral, L/3	−36	−21	45	84	4.16
TI−T2 vs TI–T1	Precentral, L/6	−42	−15	54	1471	6.58
TI–T3 vs TI–T1	Precentral, L/6	−42	−15	54	224	4.24
TI–T2 vs TI–T3	Precentral, L/9	−42	9	45	475	4.87
	Precentral, L/6	−24	−30	66	155	4.72
tDCS–T2 vs tDCS–T1	Postcentral, L/4	−21	−30	69	838	5.41
tDCS–T2 vs tDCS–T3	Precentral, L/6	−48	−3	48	264	4.31

BA, Brodmann’s area, L, left; T1: baseline; T2: during stimulation; T3: after stimulation.

## 4 Discussion

To our knowledge, this is the first study to investigate the dynamic temporal functional connectivity variability of TI. Our key finding was that TI significantly decreased the CV of dFC within M1, while increasing its mean value. This result implies that TI can dynamically reshape functional connectivity patterns within the targeted area, leading to more stable and stronger connections.

The increase in mean of dFC coupled with a decrease in CV of dFC during TI stimulation indicated an enhanced functional integration of neural activity within sensorimotor cortical regions, while demonstrating improved synchronization and heightened stability. Previous studies have discovered a link between dFC and the underlying electrophysiological mechanisms, such as fluctuations in neural oscillations across multiple frequency band (Tagliazucchi et al., 2012; Chang et al., 2013; Allen et al., 2018). The 20 Hz frequency of TI envelop may have interacted with the natural resonance frequency of the sensorimotor cortical areas, which has been reported to be around 20 Hz (Tobimatsu et al., 1999; Eusebio et al., 2009; Feurra et al., 2011). This interaction could have led to a stabilization and strengthening of beta oscillations within the motor network, resulting in increased strength and reduced variability of dFC, which is associated with enhanced motor functions including movement preparation, execution, and sensorimotor integration (Sanes and Donoghue, 1993; Baker et al., 1997; Darch et al., 2020). Moreover, 20 Hz TI may elicit frequency-specific effects in other cortical regions that interact with motor function. Specifically, the frequency-response relationship may differ due to varying neural mechanisms. For instance, alpha waves (8–12 Hz) play a crucial role in sensory-motor integration and may influence motor performance by modulating attention and perception (Klimesch, 2012). In contrast, gamma waves (30–100 Hz) are closely associated with motor preparation and execution, potentially enhancing the precision of motor control through increased synchronization among neurons (Guan et al., 2022). Research has demonstrated that oscillations at different frequencies can interact across various cortical regions, thereby affecting overall motor function (Kang et al., 2023).

In states with lower dFC variability, the brain may be able to better allocate resources and maintain efficient functioning (Cheng et al., 2018; Filippi et al., 2019; Sastry et al., 2023). Furthermore, heightened dFC strength in sensorimotor networks can lead to improved integration of sensory inputs and motor outputs, potentially resulting in more precise sensorimotor processing (Kong et al., 2021). Conversely, it is also plausible that, in certain instances, the opposite may hold true. In such cases, increased network flexibility could enhance adaptability in motor functions. For instance, a flexible network is better equipped to respond to novel motor tasks or shifting motor demands (Gonzalez-Castillo and Bandettini, 2018). Thus, it may be posited that reduced variability signifies a more rigid network, potentially constraining adaptive responses to new motor tasks or varying motor requirements (Cohen, 2018).

Additionally, the 20 Hz TI may not only influence beta oscillations (13–30 Hz) but could also impact cross-frequency interactions, particularly affecting the phase relationships between gamma waves (30–100 Hz) and alpha waves (8–12 Hz). The study by Schmidt et al. (2014) illustrates that weak electric fields can modulate the phase relationships of brain waves by influencing neuronal firing patterns. This modulation not only affects the activity within a single frequency band but may also alter interactions among different frequency bands, thereby impacting the brain’s overall functionality on a broader scale. Consequently, 20 Hz TI may enhance cognitive functions by acting on the phase relationship between gamma and alpha waves. Future research could further explore the effects of 20 Hz TI across various frequency bands, particularly concerning performance in diverse cognitive tasks. This will aid in determining whether the observed changes in dFC are confined to beta oscillations or indicative of broader alterations across frequency bands.

### 4.1 Implications for clinical applications

The findings of this study suggest that TI may have promising clinical applications in the treatment of motor-related neurological and psychiatric disorders. By dynamically reshaping functional connectivity patterns within the motor network, TI has the potential to facilitate enhanced motor performance and neuroplasticity, which could be particularly beneficial for conditions such as Parkinson’s disease and other movement disorders.

Previous studies have shown that abnormally elevated dFC variability can serve as a biomarker for various brain disorders, including Parkinson’s disease (Díez-Cirarda et al., 2018; Pang et al., 2021). The ability of TI to decrease dFC variability within the motor cortex, may indicate a more stable and integrated neural network, which could translate to improved motor function and better clinical outcomes. In patients with Parkinson’s disease, excessive beta-frequency oscillations and abnormal synchronization between neural networks are closely linked to motor symptoms (Hammond et al., 2007). Research indicates that pathological increases in dFC reflect aberrant neural synchrony, disrupting normal network dynamics (de Hemptinne et al., 2013). Reducing the dFC of these overly synchronized networks can diminish pathological beta activity, thereby improving motor function. Specifically, TI decrease abnormal dFC, and restore balance within brain networks (Polanía et al., 2011). This modulation of network dynamics is thought to may alleviate motor symptoms by reestablishing more appropriate functional connections. Moreover, the stabilization of global connectivity patterns may be a possible mechanism for global brain reorganization, which could have broader implications for the treatment of various neuropsychiatric disorders (Díez-Cirarda et al., 2018; Pang et al., 2021). By optimizing the allocation of neural resources and enhancing the efficiency of information processing, TI may contribute to improved cognitive, emotional, and behavioral outcomes in a range of clinical populations.

Overall, TI stimulation could be considered a promising tool for targeted, non-invasive neuromodulation with potential clinical applications in the treatment of motor-related disorders and beyond. Further research is needed to fully elucidate the mechanisms underlying the effects of TI stimulation on brain dynamics and to explore its clinical efficacy in diverse patient populations.

### 4.2 Comparison with tDCS

In the tDCS group, there was no significant difference on dFC variability within the M1. This indicates that tDCS cannot modulated dFC variability in the same way as TI.

This contrast in the neuromodulatory effects of the two approaches may be attributed to their distinct mechanisms of action and the ability to target specific brain regions. In current study, 20Hz-TI enables focal stimulation in specific brain regions, affecting brain function by modulating intracerebral oscillations. In contrast, the lack of significant dFC changes in the tDCS group suggests that tDCS may be less effective in penetrating. The electric fields generated by tDCS tend to decrease dramatically with depth, resulting in a lower spatial resolution and potentially reduced impact on the motor regions (Vöröslakos et al., 2018). Furthermore, there are notable differences in the mechanisms through which tDCS and TI influence motor control pathways. tDCS modifies the excitability of the entire motor pathway via direct current polarization, thereby impacting motor performance. Specifically, tDCS can enhance or inhibit neuronal activity in the motor cortex, modulating motor-related neural networks (Stagg et al., 2014). This global alteration in excitability may result in overall improvements or declines in motor control, affecting the precision and coordination of movements. In contrast, TI stimulation modulates brain oscillations by selectively targeting deeper structures within motor pathways, such as the caudate nucleus and putamen, utilizing distinct stimulation frequencies (Grossman et al., 2017; Wessel et al., 2023). By fine-tuning neural activity within motor circuit such as “cortex–basal ganglia–thalamus” circuit, TI subtly influences intrinsic cortical rhythms impacting cortical function (Wessel et al., 2023; Yang et al., 2024). This nuanced mechanism enables TI to enhance the flexibility and adaptability of motor performance, optimizing the processes underlying motor preparation and execution.

There is lack of significant dFC variability changes in the tDCS group, which may be contribute to many factors, such as the specific stimulation parameters, the individual variability in response to tDCS, and the potential need for longer stimulation durations to elicit detectable changes in dFC. Further research is warranted to better understand the mechanisms underlying the differential effects of TI stimulation and tDCS on dynamic brain connectivity.

Notably, we also observed that both tDCS and TI enhanced the mean of dFC in the M1 region, which aligns with our previous findings (Zhu et al., 2022). This suggests that although the two methods differ in their modulation of dFC variability, they both influence the overall functional connectivity in the M1 area. Further exploration of these differences may contribute to a better understanding of the mechanisms of action for different stimulation methods, thereby optimizing the application of NIBS techniques in clinical and research settings.

### 4.3 Limitations and future directions

The present study provides valuable insights of TI effects on dFC within the motor system. However, it is essential to acknowledge the limitations that need to be considered.

1) The inconsistent scan duration between different resting-state sessions (T1 and T3: 8 min each; T2: 20 min) may have influenced our results, potentially leading to a smaller coefficient of variation for T2. Additionally, potential arousal changes during the longer T2 scan should be considered. 2) The absence of both a sham condition and a systematic assessment of participants’ subjective experiences. While our focus on comparing tDCS and TI provided direct insights into their differential effects, it limited our ability to control for placebo effects and capture potential experiential differences between the interventions. Future research should address these aspects to further validate and extend our findings. 3) A lack of data collection on motor control. Future research should incorporate more behavioral measures to validate the effects of TI stimulation on motor function. This behavioral data would not only deepen our understanding of the mechanisms underlying TI but also guide the development of targeted interventions for various neurological and psychiatric conditions.

In clinical efficacy, exploring the clinical efficacy of TI stimulation in patient populations with motor-related neurological or psychiatric disorders is valuable. By directly assessing the impact of TI on motor function, cognitive performance, and other relevant clinical outcomes, researchers could further elucidate the potential of this approach for neurorehabilitation and therapeutic interventions.

In stimulation parameters, optimizing the stimulation parameters is crucial to enhance the efficiency of TI, particularly in stimulation intensity. The stimulation intensity used in the current study is referenced to low-frequency electrical stimulation (tDCS, tACS). However, TI employs a stimulation frequency of kHz, at which the resistance will be significantly smaller than that of tDCS or tACS (Zhu et al., 2022). Based on the same voltage limitation, TI can be employed at higher stimulation intensities. Moreover, individual variability represents a significant limiting factor. Attributes such as skull thickness, cortical morphology, and intrinsic oscillatory patterns may contribute to heterogeneity in responses to TES. In the future, personalized stimulation protocols can be developed by integrating neuroimaging techniques with electric field simulations based on brain structural images. This approach aims to obtain individualized optimized stimulation protocols, which is essential for enhancing the translational potential of these techniques.

## 5 Conclusion

TI stimulation can dynamically reshape functional connectivity patterns within the motor cortex. Specifically, TI targeted leading to a significant decreasing variability and increasing mean connectivity strength in dFC, potentially reflecting an interaction between the 20 Hz frequency component of the TI waveform and the natural resonance of the motor cortex. These findings position TI as a promising tool for targeted, noninvasive neuromodulation with potential clinical applications.

## Data Availability

The original contributions presented in the study are included in the article/Supplementary material, further inquiries can be directed to the corresponding author.

## Footnotes

1
http://rfmri.org/DPABI

2
http://www.fil.ion.ucl.ac.uk/spm

3
http://www.MRicro.com/MRicron
