# Supplementary material for: Syncing the brain’s networks: dynamic functional connectivity shifts from temporal interference
Source: Front Hum Neurosci. 2024 Oct 29;18:1453638. doi: 10.3389/fnhum.2024.1453638 (PMC11554487; doi:10.3389/fnhum.2024.1453638)
Supplement: Supplementary file 1 [file Data_Sheet_1.pdf]

Supplementary Material

Supplementary Table 1. brain regions showing significant differences in CV of dFC using stringent motion criteria (1.5 mm/1.5 degrees) and a 30 TR sliding window.

Comparisons	Brain regions/BA	Peak MNI coordinates			Cluster Voxels	Peak t values
		x	y	z		
Interaction effects	-	-	-	-	-	-
The main effect of group	-	-	-	-	-	-
The main effect of time	Precentral,L/6	-42	-15	54	47	4.07
tDCS-T2 vs TI-T2	Postcentral,L/4	-42	-24	57	30	3.57
TI-T1 vs TI-T2	Precentral,L/4	-39	-18	57	34	3.63
	Precentral,L/6	-30	-12	66	7	3.55

Notes. BA, Brodmann's area, L, left; T1: baseline; T2: during stimulation; T3: after stimulation.

Supplementary Table 2. Brain regions with Significant differences in CV of dFC using a 25 TR sliding window.

**Comparisons**	**Brain regions/BA**	**Peak MNI coordinates**			**Cluster Voxels**	**Peak t values**
		**x**	**y**	**z**		
**Interaction effects**	-	-	-	-	-	-
**The main effect of group**	Postcentral,L/4	-39	-21	54	10	3.21
**The main effect of time**	Precentral,L/6	-42	-15	54	49	4.15
**tDCS-T2 vs TI-T2**	Postcentral,L/4	-42	-21	57	35	3.91
**TI-T1 vs TI-T2**	Precentral,L/4	-42	-18	57	91	4.64

Notes. BA, Brodmann's area, L, left; T1: baseline; T2: during stimulation; T3: after stimulation.

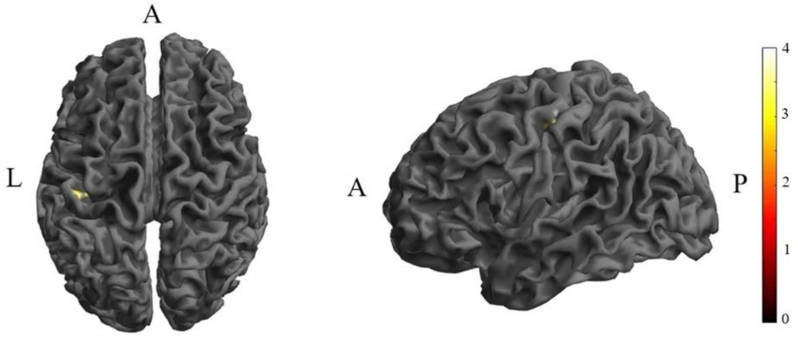

Supplementary Figure 1. Brain regions with significant CV of dFC difference between tDCS-T2 group and TI-T2 group **(applied window size: 25 TR)**. Compared with tDCS-T2 group, TI-T2 group showed significantly increased CV of dFC. The warm color denotes relatively higher values in TI-T2 group, and the color bar indicates the t-value from paired t-test between TI-T2 group and tDCS-T2 group. **Note:** CV: Coefficient of Variation; T2, during stimulation; A, anterior; L, left; P, posterior.

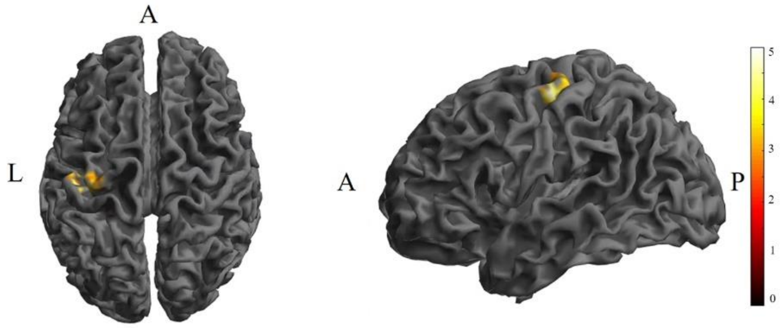

Supplementary Figure 2. Brain regions with significant CV of dFC difference between TI-T1 group and TI-T2 group **(applied window size: 25 TR)**. Compared with TI-T1 group, TI-T2 group showed significantly increased CV of dFC. The warm color denotes relatively higher values in TI-T2 group, and the color bar indicates the t-value from paired t-test between TI-T2 group and tDCS-T2 group. **Note:** CV: Coefficient of Variation; T1, at baseline; T2, during stimulation; A, anterior; L, left; P, posterior.

Supplementary Table 3. Brain regions with Significant differences in mean of dFC using a 25 TR sliding window.

Comparisons	Brain regions/BA	Peak MNI coordinates			Cluster Voxels	Peak t values
		x	y	z		
**Interaction effects**	-	-	-	-	-	-
**The main effect of group**	Postcentral,L/3	-45	-24	60	33	3.8
**The main effect of time**	Postcentral,L/4	-21	-30	69	1423	6.61
**TI-T2 vs tDCS-T2**	Postcentral,L/3	-48	-18	60	88	4.17
**TI-T2 vs TI-T1**	Precentral,L/6	-42	-15	54	1473	6.59

**TI-T3 vs TI-T1**	Precentral,L/6	-42	-15	54	208	4.26
**TI-T2 vs TI-T3**	Precentral,L/9	-42	9	45	482	4.85
	Precentral,L/6	-24	-30	66	159	4.72
**tDCS-T2 vs tDCS-T1**	Postcentral,L/4	-21	-30	69	826	5.37
**tDCS-T2 vs tDCS-T3**	Precentral,L/6	-48	-3	48	273	4.28

Notes. BA, Brodmann's area, L, left; T1: baseline; T2: during stimulation; T3: after stimulation.

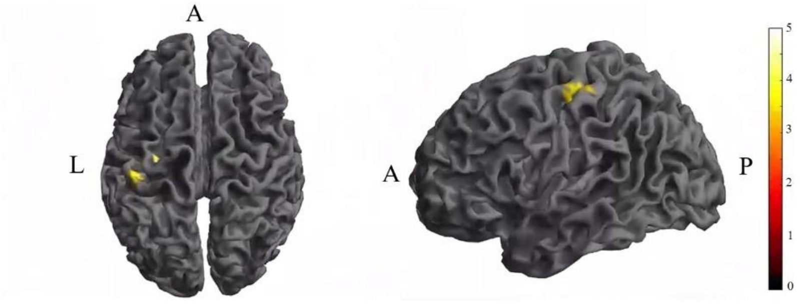

Supplementary Figure 3. Brain regions with significant mean of dFC difference between tDCS-T2 group and TI-T2 group **(applied window size: 25 TR)**. Compared with tDCS-T2 group, TI-T2 group showed significantly increased mean of dFC. The warm color denotes relatively higher values in TI-T2 group, and the color bar indicates the t-value from paired t-test between TI-T2 group and tDCS-T2 group. **Note:** T2, during stimulation; A, anterior; L, left; P, posterior.

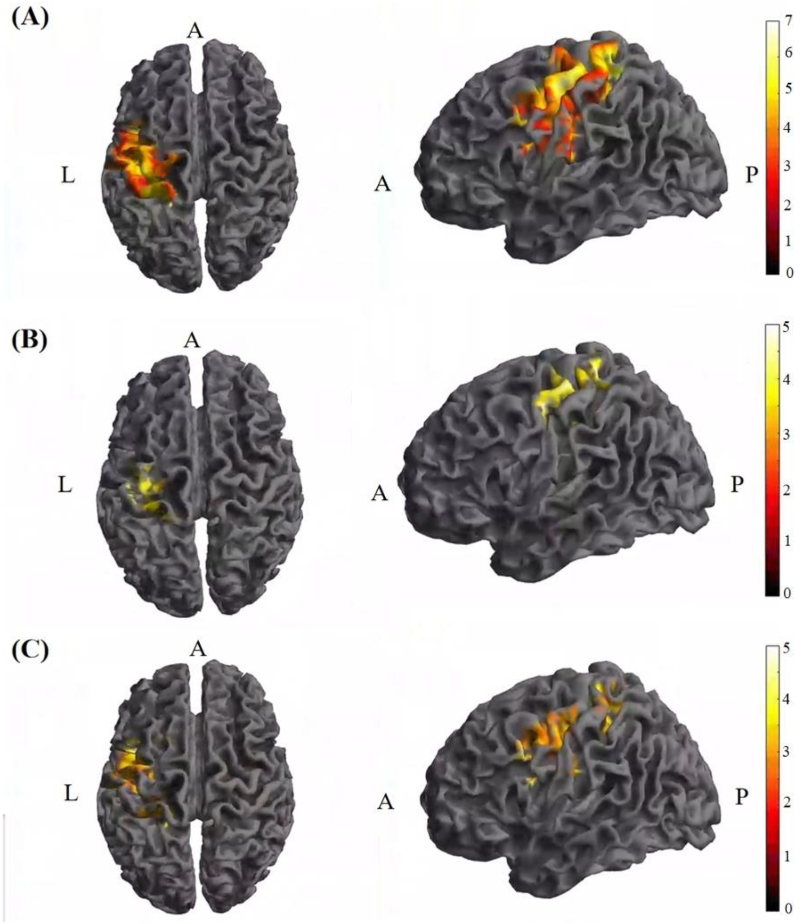

Supplementary Figure 4. (A) Brain regions with significant mean of dFC difference between TI-T2 group and TI-T1 group. Compared with TI-T1 group, TI-T2 group showed significantly increased mean of dFC. **(B)** Brain regions with significant mean of dFC difference between TI-T3 group and TI-T1 group. Compared with TI-T1 group, TI-T3 group showed significantly increased mean of dFC. **(C)** Brain regions with significant mean of dFC difference between TI-T2 group and TI-T3 group. Compared with TI-T3 group, TI-T2 group showed significantly increased mean of dFC **(applied window size: 25 TR)**. The warm color denotes relatively higher values, and the color bar indicates the t-value from paired t-test. **Note:** T1, at baseline; T2, during stimulation; T3, post stimulation; A, anterior; L, Left; P, posterior.

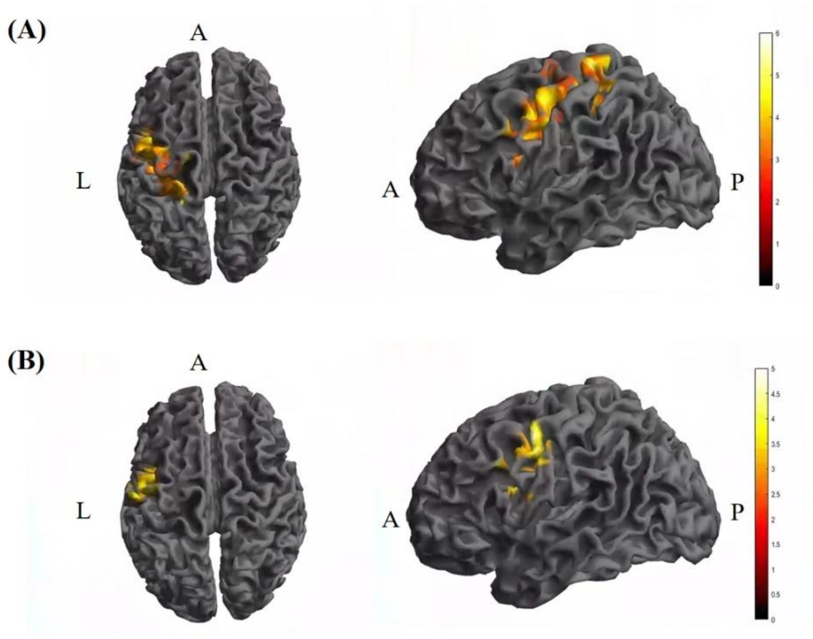

Supplementary Figure 5. (A) The significant differences in brain regions in mean of dFC between tDCS-T2 and tDCS-T1. **(B)** The significant differences in brain regions in mean of dFC between tDCS-T2 and tDCS-T3 **(applied window size: 25 TR)**. **Note:** The color bar represents the T value. T1, at baseline; T2, during stimulation; T3, post stimulation; A, anterior; L, Left; P, posterior.

Supplementary Table 4. Brain regions with Significant differences in CV of dFC using a 35 TR sliding window.

**Comparisons**	**Brain regions/BA**	**Peak MNI coordinates**			**Cluster Voxels**	**Peak t values**
		**x**	**y**	**z**		
**Interaction effects**	-	-	-	-	-	-
**The main effect of group**	Postcentral,L/3	-48	-24	51	12	3.25
**The main effect of time**	Precentral,L/6	-42	-15	54	51	4.11
**tDCS-T2 vs TI-T2**	Postcentral,L/3	-30	-27	51	30	4.06
**TI-T1 vs TI-T2**	Precentral,L/4	-42	-18	57	87	4.56

Notes. BA, Brodmann’s area, L, left; T1: baseline; T2: during stimulation; T3: after stimulation.

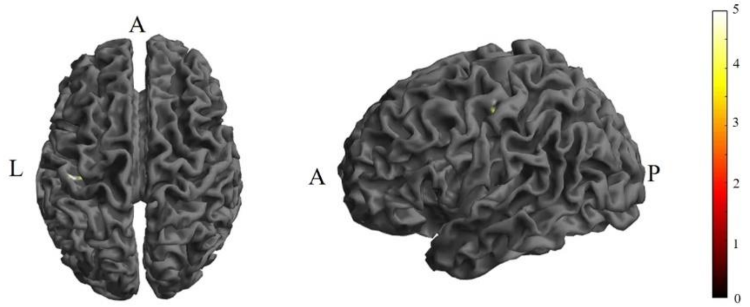

Supplementary Figure 6. Brain regions with significant CV of dFC difference between tDCS-T2 group and TI-T2 group **(applied window size: 35 TR)**. Compared with tDCS-T2 group, TI-T2 group showed significantly increased CV of dFC. The warm color denotes relatively higher values in TI-T2 group, and the color bar indicates the t-value from paired t-test between TI-T2 group and tDCS-T2 group. **Note:** CV: Coefficient of Variation; T2, during stimulation; A, anterior; L, left; P, posterior.

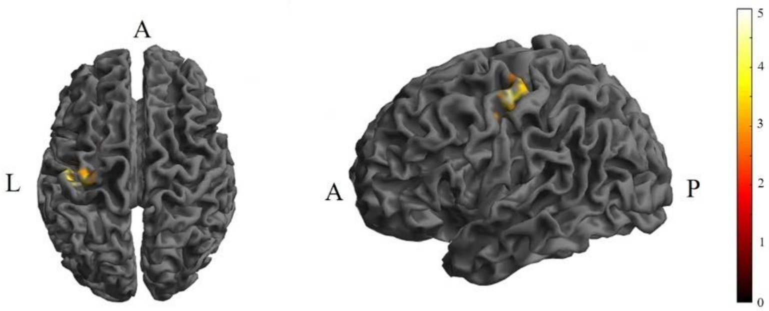

Supplementary Figure 7. Brain regions with significant CV of dFC difference between TI-T1 group and TI-T2 group **(applied window size: 35 TR)**. Compared with TI-T1 group, TI-T2 group showed significantly increased CV of dFC. The warm color denotes relatively higher values in TI-T2 group, and the color bar indicates the t-value from paired t-test between TI-T2 group and tDCS-T2 group. **Note:** CV: Coefficient of Variation; T1, at baseline; T2, during stimulation; A, anterior; L, left; P, posterior.

Supplementary Table 5. Brain regions with Significant differences in mean of dFC using a 35 TR sliding window.

Comparisons	Brain regions/BA	Peak MNI coordinates			Cluster Voxels	Peak t values
		x	y	z		
**Interaction effects**	-	-	-	-	-	-
**The main effect of group**	Postcentral,L/3	-45	-24	60	41	3.75
**The main effect of time**	Precentral,L/6	-42	-12	54	1426	6.60
**TI-T2 vs tDCS-T2**	Postcentral,L/3	-48	-15	60	81	4.14
**TI-T2 vs TI-T1**	Precentral,L/6	-39	-15	54	1472	6.58
**TI-T3 vs TI-T1**	Precentral,L/6	-42	-15	54	225	4.20
**TI-T2 vs TI-T3**	Precentral,L/44	-42	9	39	449	4.90
	Precentral,L/6	-24	-30	66	150	4.70
**tDCS-T2 vs tDCS-T1**	Postcentral,L/4	-21	-30	69	883	5.42
**tDCS-T2 vs tDCS-T3**	Precentral,L/6	-48	-3	48	269	4.32

Notes. BA, Brodmann’s area, L, left; T1: baseline; T2: during stimulation; T3: after stimulation.

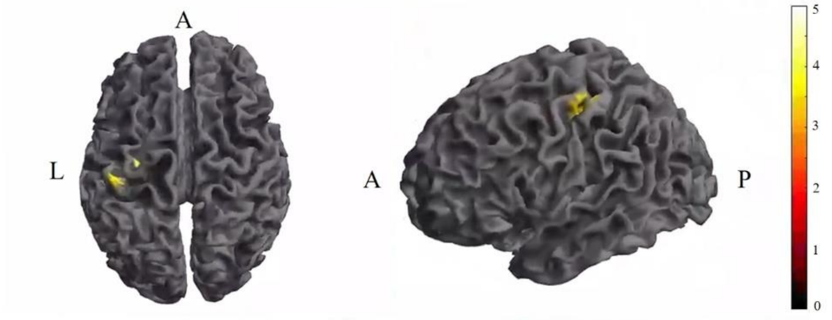

Supplementary Figure 8. Brain regions with significant mean of dFC difference between tDCS-T2 group and TI-T2 group **(applied window size: 35 TR)**. Compared with tDCS-T2 group, TI-T2 group showed significantly increased mean of dFC. The warm color denotes relatively higher values in TI-T2 group, and the color bar indicates the t-value from paired t-test between TI-T2 group and tDCS-T2 group. **Note:** T2, during stimulation; A, anterior; L, left; P, posterior.

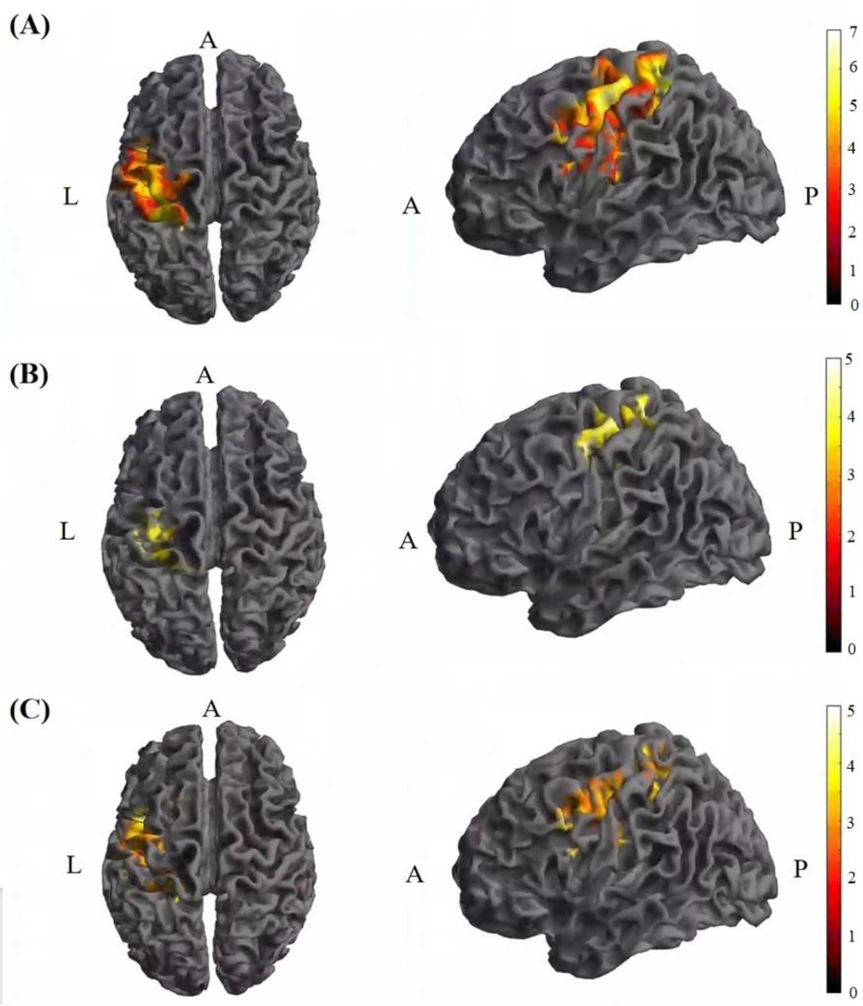

Supplementary Figure 9. (A) Brain regions with significant mean of dFC difference between TI-T2 group and TI-T1 group. Compared with TI-T1 group, TI-T2 group showed significantly increased mean of dFC. **(B)** Brain regions with significant mean of dFC difference between TI-T3 group and TI-T1 group. Compared with TI-T1 group, TI-T3 group showed significantly increased mean of dFC. **(C)** Brain regions with significant mean of dFC difference between TI-T2 group and TI-T3 group. Compared with TI-T3 group, TI-T2 group showed significantly increased mean of dFC **(applied window size: 35 TR)**. The warm color denotes relatively higher values, and the color bar indicates the t-value from paired t-test. **Note:** T1, at baseline; T2, during stimulation; T3, post stimulation; A, anterior; L, Left; P, posterior.

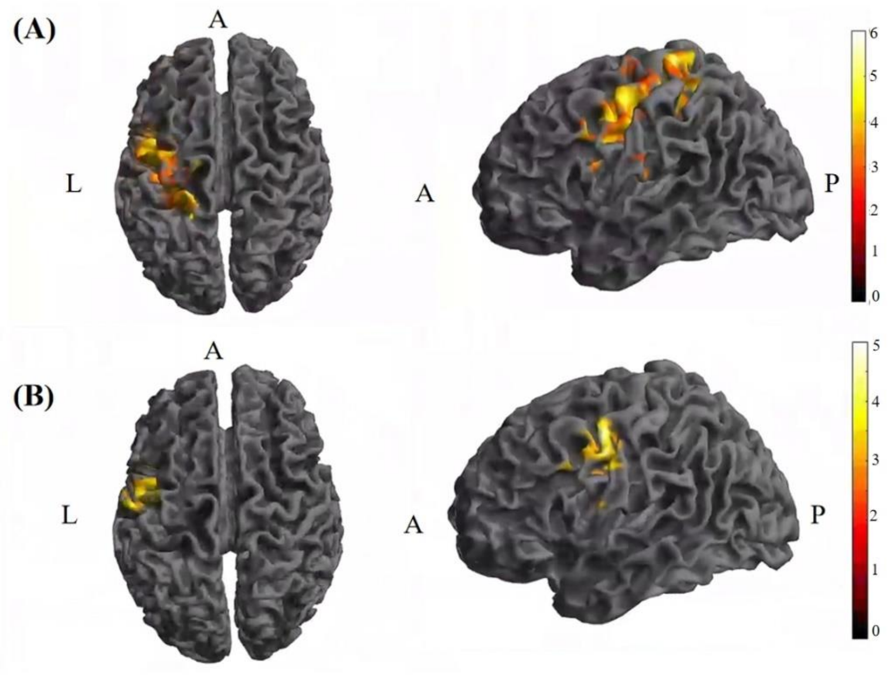

Supplementary Figure 10. (A) The significant differences in brain regions in mean of dFC between tDCS-T2 and tDCS-T1. **(B)** The significant differences in brain regions in mean of dFC between tDCS-T2 and tDCS-T3 **(applied window size: 35 TR)**. **Note:** The color bar represents the T value. T1, at baseline; T2, during stimulation; T3, post stimulation; A, anterior; L, Left; P, posterior.
